# Comparing clinical presentations, treatments and outcomes of hepatocellular carcinoma due to hepatitis C and non-alcoholic fatty liver disease

**DOI:** 10.1093/qjmed/hcw151

**Published:** 2016-09-10

**Authors:** Nwe Ni Than, Anwar Ghazanfar, James Hodson, Nadeem Tehami, Chris Coldham, Hynek Mergental, Derek Manas, Tahir Shah, Philip N. Newsome, Helen Reeves, Shishir Shetty

**Affiliations:** From the 1 Liver Unit, University Hospitals Birmingham NHS Trust, Birmingham, Edgbaston, UK; 2 National Institute for Health Research (NIHR) Birmingham Liver Biomedical Research Unit and Centre for Liver Research, University of Birmingham, Birmingham, UK; 3 Liver Unit, Newcastle Upon Tyne NHS Foundation Trust, Newcastle Upon Tyne, UK

## Abstract

**Introduction:** Hepatocellular carcinoma (HCC) is increasing in incidence in the UK and globally. Liver cirrhosis is the common cause for developing HCC. The common reasons for liver cirrhosis are viral hepatitis C (HCV), viral hepatitis B and alcohol. However, HCC caused by non-alcoholic fatty liver disease (NAFLD)-cirrhosis is now increasingly as a result of rising worldwide obesity.

**Aim**: To compare the clinical presentation, treatment options and outcomes of HCC due to HCV and NAFLD patients.

**Methods:** Data were collected from two liver transplant centres in the UK (Birmingham and Newcastle upon Tyne) between 2000 and 2014. We compared 275 patients with HCV-related HCC against 212 patients with NAFLD- related HCC.

**Results:** Patients in the NAFLD group were found to be significantly older (*P* < 0.001) and more likely to be Caucasian (*P* < 0.001). They had lower rates of cirrhosis (*P* < 0.001) than those in HCV-HCC group. The NAFLD group presented with significantly larger tumours (*P* = 0.009), whilst HCV patients had a higher alpha fetoprotein (*P* = 0.018). NAFLD patients were more commonly treated with TACE (*P* = 0.005) than the HCV patients, whilst the HCV group were significantly more likely to be transplanted (*P* < 0.001). In patients selected for liver transplantation, 5-year survival rates in NAFLD were not significantly different from HCV-HCC (44 and 56% respectively, *P* = 0.102).

**Conclusion:** In this study, NAFLD patients presented with larger tumours that were less likely to be amenable to curative therapy, as compared with HCV patients. Despite this disadvantage, patients with NAFLD had similar overall survival compared to patients with HCV.

## Introduction

Hepatocellular carcinoma (HCC), constituting 70–90% of cases of primary liver cancer, is the fifth most common cause of cancer in Europe and a life threatening complication of cirrhosis^1^ European epidemiological data show that there are 1–13 new cases of HCC and 1–10 deaths per 100 000 inhabitants per year.^1^ A study from the USA conducted in 1999 showed increased incidence of HCC in the past two decades.^2^ Without any treatment, HCC has a very poor prognosis, with a 5-year survival rate of around 5%.^1^ Chronic viral hepatitis, caused by the hepatitis B virus (HBV) and hepatitis C virus (HCV), is known to be an additional risk factor and there is gathering evidence that HCC associated with these infections have differences in their molecular signatures compared to HCC with other causes of cirrhosis.^3^ Recently, there has been a dramatic progress in the therapeutic options for HBV and HCV infection, leading to effective viral suppression in HBV and high rates of cure for HCV.^4^ This will significantly alter the epidemiology of HCC in the future with likely decline in viral associated cirrhosis and HCC. Non-alcoholic fatty liver disease (NALFD) on the other hand is an emerging public health problem that is an increasing cause of cirrhosis and HCC.^5^^,^^6^

NAFLD-associated HCC usually occurs at a more advanced age (4–6 years older) than HCC caused by cirrhosis of other aetiologies.^7^ In some patients with NAFLD, HCC can occur without underlying features of liver cirrhosis and a study from USA showed only 46% of NAFLD- and non-alcoholic steatohepatitis (NASH)-related HCC patients have underlying cirrhosis.^8^ Increased body mass is associated with increased risk of all cancers, including liver cancer.^9^ A population study from Sweden showed threefold higher risk of HCC in obese patients^10^ and a Danish study further confirmed twofold increased in liver cancer incidence in obese subjects compared with non-obese subjects.^11^ A recent study from the USA showed that compared with normal weight individuals, obese individuals had a 2.4-fold increased risk of liver cancer (OR = 2.38, 95% CI: 1.68–3.36), and overweight individuals had a 32% increased risk (OR = 1.32, 95% CI: 1.03–1.70).^12^

Diabetes has been found to increase the risk of developing chronic liver disease and HCC^13^ and in a recent systematic review of 13 case-control studies, diabetic subjects were found to have a twofold increase in the risk of HCC compared with a cohort of patients without diabetes.^14^^,^^15^ Although studies have been conducted to assess the incidence and mortality of HCC in patients with NAFLD compared with viral hepatitis C related HCC,^16–20^ there are limited data on outcomes of patients who had liver transplantation for NAFLD associated HCC.

With the change in the management of viral hepatitis and increasing incidence of NAFLD, it is useful to compare the presentation and outcome of HCC in patients with HCV and NAFLD in a cohort from the UK, in order to help devise future strategies in surveillance and management of HCC. The aim of this study was to compare baseline demographic features, tumour characteristics and the clinical outcomes of patients with NAFLD-associated HCC compared to those with HCV-associated HCC.

## Methods

### Ethical statement

This was a retrospective study and was registered with NHS trust audit departments in both units (Newcastle upon Tyne and Birmingham).

Data were retrospectively collected from all adult patients (≥18 years) with HCC secondary to HCV or NAFLD who were referred to two liver transplant centres in UK (Birmingham and Newcastle Upon Tyne liver units) between 2000 and 2014. Patients were excluded if they had both co-existing NAFLD and HCV.

HCV infection was identified by antibody testing, before being confirmed with polymerase chain reaction and viral load tests. The diagnosis of NAFLD was made when there was evidence of liver steatosis on imagining, or the histologic features of NASH, when available, or cryptogenic cirrhosis in the presence of metabolic syndrome and without a history of significant alcohol intake. Metabolic syndrome was defined following the National Cholesterol Education Program Adult Treatment Plan III guidelines. Patients with cirrhosis were identified based on histological features of cirrhosis and/or radiological evidence of cirrhosis in the context of portal hypertension (ascites, variceal bleeding, thrombocytopenia or hepatic encephalopathy).

Demographic details (age, gender and ethnicity), alcohol excess (defined as more than 21 units in men and 14 units in women), presence of diabetes mellitus (DM) and body mass index (BMI) were extracted from the patient’s records. Model for end stage liver disease (MELD) and albumin levels were collected at the time of diagnosis.

Patients who had ultrasound scans suggesting HCC received further radiological tests, generally magnetic resonance imaging scans, with diagnosis made where a typical vascular pattern of HCC was detected. Details of tumour characteristics such as size, numbers, location, vascular invasions and distant organ metastasis were collected from radiology reports. Alpha-feto protein (AFP) levels were collected at the time of diagnosis of HCC.

### Statistical analysis

Normally distributed continuous variables were reported as means and standard deviations, which were compared between groups using independent samples *t*-tests. Non-parametric variables were reported as medians with either the range or quartiles, with comparisons performed using Mann–Whitney tests. For the categorical variables, Fisher’s exact tests were used.

Kaplan-Meier curves were then used to compare survival between NAFLD and HCV patients, both from diagnosis and from transplant with comparisons between the disease groups made using log-rank tests. A Kaplan-Meier approach was also used in the comparison of transplant rates, with patients censored at death, in order to account for the timings of transplants and variable lengths of follow up across patients.

Multivariable cox regression models were then produced, to test whether the differences in survival from diagnosis between the two diseases were independent of other potentially confounding factors. The disease group was entered into the model and a forward stepwise approach was used to add independent predictors of patient survival. Any factors with >10% missing data that were not found to be significant in this model were then excluded, and the model re-run, in order to maximize the available sample size.

All analyses were performed using IBM SPSS Statistics 22 (IBM Corp. Armonk, NY). Missing data were excluded on a per-analysis basis and *P* < 0.05 was deemed to be indicative of statistical significance throughout.

## Results

### Baseline characteristics

A total of 488 patients with HCC caused by either HCV or NAFLD presented to the liver transplant centres in Birmingham and Newcastle upon Tyne between 2000 and 2014. One patient was excluded due to the presence of co-existing HCV and NAFLD in this particular patient. Among the final 487 patients (Birmingham, *n* = 290 and Newcastle upon Tyne, *n* = 197), 275 patients had HCC due to HCV and 212 patients had HCC secondary to NAFLD. The median duration of follow up was 1 year (Range: 0, 14 years). 20/275 HCV patients (7%) had co-infection with viral hepatitis B infection and 2/275 (0.7%) patients had underlying co-existing human immunodeficiency virus (HIV) infection.

The mean age of the entire population was 63 years (SD 11.0, range 30, 91 years) of which 390 were male (80%) and 406 were Caucasian (83%). Patients with NAFLD were significantly older than those with HCV at the time of HCC diagnosis (mean age of 69.6 vs. 58.6, respectively, *P* < 0.001). Patients with NAFLD were also more likely to be Caucasian (98 vs. 72%, *P* < 0.001), had higher BMI (mean 32.3 vs. 26.5, *P* < 0.001) and were more likely to have T2DM (72 vs. 24%, *P* < 0.001) than those who had HCV. The rate of excess alcohol consumption (>21 units in men and  >14 units in women) were similar in the two groups (14% in NAFLD vs. 18% in HCV, *P* = 0.215). At the time of HCC diagnosis, patients with HCV were more likely to be cirrhotic (99 vs. 87%, *P* < 0.001) than those with NAFLD.

The median MELD at diagnosis of HCC was not found to differ significantly between the two groups (9 in HCV vs. 10 in NAFLD, *P* = 0.142). Albumin levels were significantly higher in NAFLD patients than in HCV (mean 39.3 vs. 37.2 g/l, *P* = 0.006). Among 487 patients, 101 patients (21%) (80 HCV and 21 NAFLD) were transplanted.

**Figure 1:**
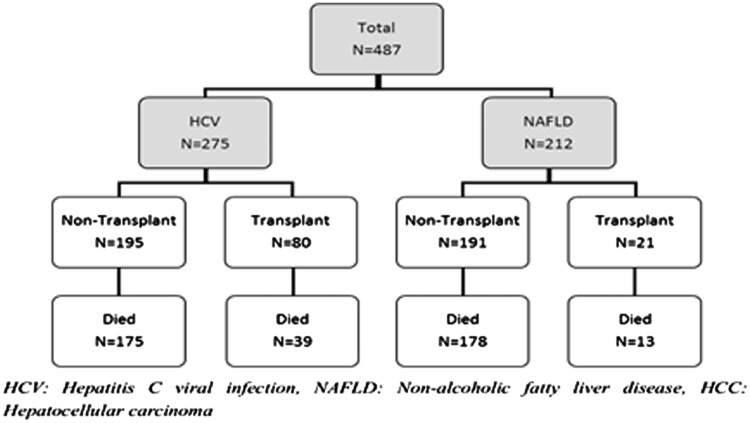
Flowchart of cohort of patients with HCC.

Figure 1 and Table 1 summarized the overall patients’ characteristics and their demographics.

**Table 1:** Baseline demographics of overall and transplanted cohort of patients

Total cohort of patients	Valid *n*	HCV *(n* *=* *275)*	NAFLD * (n* *=* *212)*	*P-*value
Age^a^	487	58.6 (10.2)	69.6 (8.7)	<0.001*^a^
BMI^a^	336	26.5 (5.2)	32.3 (5.6)	<0.001*^a^
Gender (Male)	487	219 (79.6%)	171 (80.7%)	0.820
Ethnicity	487			<0.001*
*White*		198 (72.0%)	208 (98.1%)	
*Asian*		66 (24.0%)	3 (1.4%)	
*Black/Mixed*		11 (4.0%)	1 (0.5%)	
DM (type 2)	487	65 (23.6%)	153 (72.2%)	<0.001*
Alcohol Excess	487	50 (18.2%)	29 (13.7%)	0.215
Cirrhotic	487	271 (98.5%)	185 (87.3%)	<0.001*
MELD^b^	287	9 (7 – 11)	10 (8 – 13)	0.142^b^
Albumin^a^	288	37.2 (6.0)	39.3 (5.6)	0.006*^a^

**Transplanted cohort of patients**	**Valid *n***	**HCV *(n=80)***	**NAFLD *(n=21)***	*P*-value

Age^a^	101	54.0 (7.2)	58.9 (5.5)	0.005*^a^
BMI^a^	98	26.9 (4.5)	32.1 (5.0)	<0.001*^a^
Gender (Male)	101	69 (86%)	18 (86%)	1.000
Ethnicity	101			0.012*
White		58 (73%)	21 (100%)	
Asian		20 (25%)	0 (0%)	
Black/Mixed		2 (3%)	0 (0%)	
DM (type 2)	101	27 (34%)	16 (76%)	<0.001*
Alcohol Excess	101	10 (13%)	6 (29%)	0.094
MELD^b^	101	8 (7–11)	10 (8–15)	0.024*^b^
Albumin^a^	101	37.9 (5.8)	35.9 (7.3)	0.177^a^

Data reported as ‘*n* (%)’, with *P*-values from Fisher’s exact test, unless stated otherwise.

^a^Data reported as ‘Mean (SD)’, with *P*-values from independent samples *t*-tests.

^b^Data reported as ‘Median (Quartiles)’, with *P-*values from Mann–Whitney tests.

* Significant at *P* < 0.05.

### Previous HCV treatment

In patients with chronic hepatitis C infection, 74/275 patients (27%) were previously treated with PEGylated interferon and ribavirin and among them, 20 patients (27%) responded to the combination treatment.

### HCC characteristics in both patients’ groups

The HCC characteristics for all patients were documented in Table 2. AFP levels were found to be higher in HCV (median of 32 vs. 12, *P* = 0.018) than in patients with NAFLD. The majority of HCCs were found in the right lobe of the liver (59% in NAFLD vs. 63% in HCV), followed by both lobes and the left lobe, with distribution of HCCs being similar in the two groups (*P* = 0.891). Patients with NAFLD had larger tumours, with 39% of cases being 5 cm or more, compared with 26% in HCV (*P* = 0.009). 74% of NAFLD patients and 76% of HCV patients presented with less than 3 tumours at the time of first presentation. 18% of patients from both groups had three to five lesions and <10% of patients had more than five lesions at time of presentation. 14% of patients in both groups had evidence of either macro or micro vascular invasion, either from radiological examination or histologically post-liver transplantation. In the whole cohort, lymph node metastasis was noted in 2% of cases and distant organ metastasis was found in 5% of cases at diagnosis.

**Table 2:** HCC comparisons between the disease groups (overall and transplanted cohort)

Total cohort of patients	Valid *n*	HCV *(n =* *275)*	NAFLD * (n* *=* *212)*	*P-*value
Location of HCC	387			0.891
Right		145 (62.5%)	92 (59.4%)	
Left		30 (12.9%)	22 (14.2%)	
Both		48 (20.7%)	36 (23.2%)	
Others		9 (3.9%)	5 (3.2%)	
Largest HCC Size (cm)	455			0.009*
<2.0		40 (15.6%)	23 (11.6%)	
2.0–4.9		151 (59.0%)	99 (49.7%)	
5.0+		65 (25.4%)	77 (38.7%)	
Number of HCC	486			0.520
0–2		208 (75.9%)	156 (73.6%)	
3–5		49 (17.9%)	37 (17.5%)	
>5		16 (6.2%)	19 (9.0%)	
AFP^a^	458	32 (8 - 199)	12 (4 - 212)	0.018*^,^^a^
Vascular Invasion^b^	487	39 (14.2%)	29 (13.7%)	0.896
Lymph nodes	487	7 (2.5%)	4 (1.9%)	0.763
Distant Organ Mets	487	11 (4.0%)	13 (6.1%)	0.298


Transplanted cohort of patients	Valid *n*	HCV *(n =* *80)*	NAFLD * (n* *=* *21)*	*P-*value
Location of HCC	86			0.757
Right		39 (57%)	9 (50%)	
Left		5 (7%)	1 (6%)	
Both		17 (25%)	7 (39%)	
Others		7 (10%)	1 (6%)	
Largest HCC Size (cm)	101			0.150
<2.0		18 (23%)	8 (38%)	
2.0–4.9		61 (76%)	12 (57%)	
5.0+		1 (1%)	1 (5%)	
Number of HCC	101			0.471
0–2		61 (76%)	18 (86%)	
3–5		16 (20%)	2 (10%)	
>5		3 (4%)	1 (5%)	
AFP^a^	99	11 (5–39)	5 (3–8)	0.006*^,^^a^


Data reported as ‘*n* (%)’, with *P*-values from Fisher’s exact test, unless stated otherwise.

^a^Data reported as ‘Median (Quartiles)’, with *P-*values from Mann–Whitney tests.

^b^indicates either radiological or histological evidence.

* Significant at *P* < 0.05.

### HCC treatments in both patients’ groups

The summary of HCC treatments in both groups were documented in Table 3. Patients with NAFLD were more likely to be treated with trans-arterial chemoembolization (TACE) (41 vs. 28%, *P* = 0.004). Radiofrequency ablation was used in 15% of patients, followed by liver resection in 3–4% of cases. Sorafenib therapy was given in a similar proportion of HCV and NAFLD cases (5.5 vs. 7.1%, *P* = 0.569). During the follow up period of 14 years, HCV patients were more likely to undergo liver transplantation (29 vs. 9.9%, *P* < 0.001). Due to the varying lengths of follow up, transplantation rates were estimated by a Kaplan-Meier approach.

**Table 3:** Treatment comparisons between the disease groups

	Valid *n*	HCV *(n* *=* *275)*	NAFLD *(n* *=* *212)*	*P*-value
Radio frequency ablation (RFA)	487	42 (15.4%)	29 (13.7%)	0.698
Trans arterial chemo-embolization (TACE)	487	78 (28.4%)	87 (41.0%)	0.004*
Liver resection	487	8 (2.9%)	9 (4.2%)	0.463
Percutaneous ethanol Injection (PEI)	487	9 (3.3%)	4 (1.9%)	0.407
Sorafenib therapy	487	15 (5.5%)	15 (7.1%)	0.569
Liver transplantation rates over 14 years follow up	487	80 (29.1%)	21 (9.9%)	**<0.001**
Liver transplantation by 5 years^a^	487	56.3%	16.8%	<0.001*^a^

Data reported as ‘*n* (%)’, with *P*-values from Fisher’s exact test.

^a^Kaplan-Meier estimated rate, censored at death, with *P*-values from log-rank test.

* Significant at *P* < 0.05.

### Survival data

**Figure 2:**
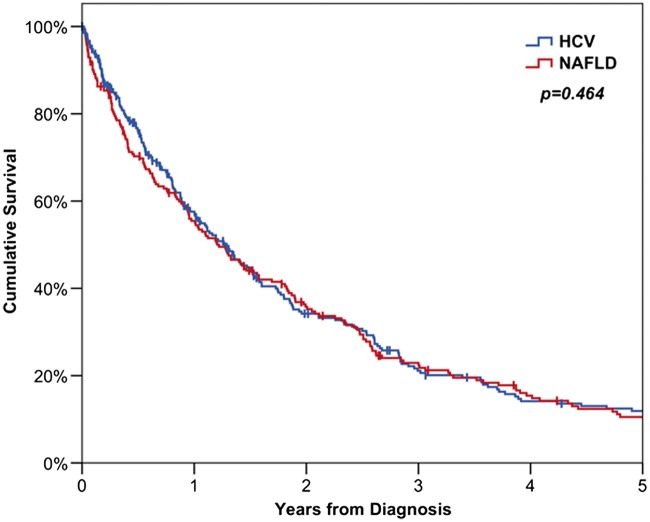
Kaplan-Meier curves for overall survival of patients with HCC. HCV, Hepatitis C viral infection; NAFLD, non-alcoholic fatty liver disease.

The survival outcomes for the two groups are reported in Table 4. Overall survival was similar in the two groups, with rates at 3 years from diagnosis were 21% for HCV and 23% for NAFLD (*P* = 0.464, Figure 2).

**Table 4:** Survival rates of patients with HCC

	*n*	1 year	3 years	5 years	*P*-values
Overall survival from diagnosis					***0.464***
HCV	*275*	57.6% (3.2%)	21.1% (2.8%)	11.9% (2.3%)	
NAFLD	*212*	55.5% (3.5%)	22.9% (3.0%)	10.5% (2.3%)	
Survival from diagnosis for non-OLT patients					***0.157***
HCV	*195*	46.2% (3.7%)	10.1% (2.3%)	3.7% (1.5%)	
NAFLD	*191*	51.6% (3.7%)	18.2% (2.9%)	6.8% (2.0%)	

OLT, orthoptic liver transplantation; HCV, hepatitis C viral infection; NAFLD, non-alcoholic fatty liver disease.

Data reported as ‘Kaplan-Meier estimate (SE)’ with *P-*values from log-rank tests.

Since there were large differences in the demographics of the two groups, a multivariable analysis was then performed (Table 5). All of the factors in Tables 1–3 were considered for inclusion in the model. The analysis found cirrhosis (*P* = 0.008), increasing HCC size (*P* < 0.001) and AFP (*P* < 0.001) and vascular invasion (*P* = 0.001) to be significant predictors of shorter survival. Treatment with RFA (*P* = 0.026), liver resection (*P* = 0.041) Sorafenib (*P* < 0.001) and transplant (*P* = 0.003) were all found to significantly lengthen survival. After accounting for these factors, the difference in survival between the two disease groups remained non-significant (HR: 1.22, 95% CI: 0.97–1.54, *P* = 0.084).

**Table 5:** Multivariable analysis of survival in all patients

	HR (95% CI)	*P*-value
Disease (NAFLD)	1.22 (0.97–1.54)	0.084
**Cirrhotic**	**1.91 (1.18–3.08)**	0.008*
**Largest HCC Size (cm)**		<0.001*
<2.0		
2.0-4.9	1.84 (1.28–2.63)	<0.001*
5.0+	2.68 (1.77–4.04)	<0.001*
**AFP**		<0.001*
<5		
5–24	1.34 (0.98–1.83)	0.068
25–249	1.80 (1.32–2.47)	<0.001*
250+	2.14 (1.53–3.01)	<0.001*
**Vascular invasion**	**1.79 (1.26–2.53)**	0.001*
**Radiofrequency ablation**	**0.69 (0.50–0.96)**	0.026*
**Liver resection**	**0.51 (0.27–0.97)**	0.041*
**Sorafenib therapy**	**0.31 (0.20–0.48)**	<0.001*
**Transplanted**^a^	**0.55 (0.37–0.82)**	0.003*

Results from a multivariable cox regression model, using a forward stepwise entry procedure. The disease group was forced into the model, and factors in Tables 1–3 were considered for inclusion as additional confounders. The initial model did not identify any of the factors with >10% missing data as significant predictors of survival, hence these were excluded to maximize the available sample size, and the model re-run. The final model was based on the *n* = 355 cases with data available for all of the factors considered.

HR, hazard ratio.

^a^Treated as a time-dependent covariate, in order to account for the effect of survivor bias.

* Significant at *P* < 0.05.

Univariable analyses of the factors considered are also reported in Supplementary Table S1.

### Sub-group analysis

#### Transplanted cohort

**Figure 3:**
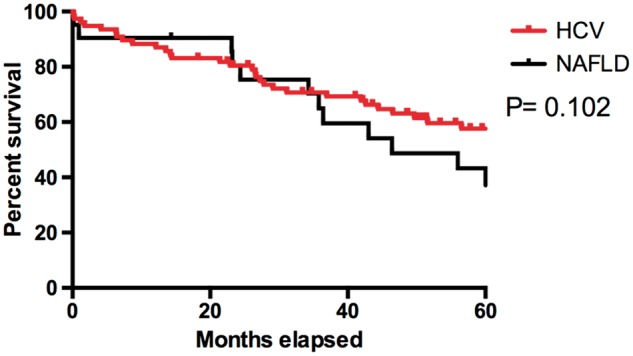
Kaplan-Meier curves for post-transplant survival of patients receiving transplants for HCC. HCV, Hepatitis C viral infection; NAFLD, non-alcoholic fatty liver disease.

Analysis was also performed on the subgroup of patients that received liver transplants during the follow up period (*n* = 101), all of whom were cirrhotic at the time of listing. Tables 1 and 2 summarize the baseline characteristics and tumour characteristics of the transplanted cohort, as well as comparisons between HCV and NAFLD. These comparisons returned similar results to the analysis of the whole patient cohort. The only exceptions were the analyses of MELD, which was found to be significantly higher in the transplanted NAFLD cohort (*P* = 0.024), and the comparisons of Albumin and tumour size, which became non-significant on account of the smaller sample size. The post-transplant survival for patients in both groups were shown in Figure 3, with 5-year survival rates in NAFLD-HCC were not significantly different from HCV-HCC (44 and 56%, respectively, *P* = 0.102). The survival for patients with HCC who did not receive liver transplantation (*n* = 386) was poor, with only 4% of HCV and 7% of NAFLD patients surviving 5 years from diagnosis.

#### Comparison between cirrhotic and non-cirrhotic patients

Patients without cirrhosis were significantly older (mean age of 72 ± 11 vs. 63 ± 11 years, *P* < 0.001) compared to cirrhotic patients. DM was significantly more common in non-cirrhotic patients (71 vs. 43%, *P* = 0.003) but the two groups had similar BMIs (mean 30 ± 5 vs. 29 ± 5, *P* = 0.300). MELD was also similar in the two groups (*P* = 0.140) but albumin was significantly higher in non-cirrhotic patients (mean 43.3 ± 3.5 vs. 37.6 ± 6.0, *P* = 0.005). The locations and the numbers of HCCs were similar in both groups (*P* = 0.676, 0.645). AFP was similar between the two groups (*P* = 0.902). Non-cirrhotic patients were more likely to undergo liver resections (26 vs. 2%, *P* < 0.001), with patients that received liver resection (*n* = 17) having a significantly greater 5-year survival rate of 27% compared wtih 11% in non- liver resected patients (*P* = 0.001).

**Figure 4:**
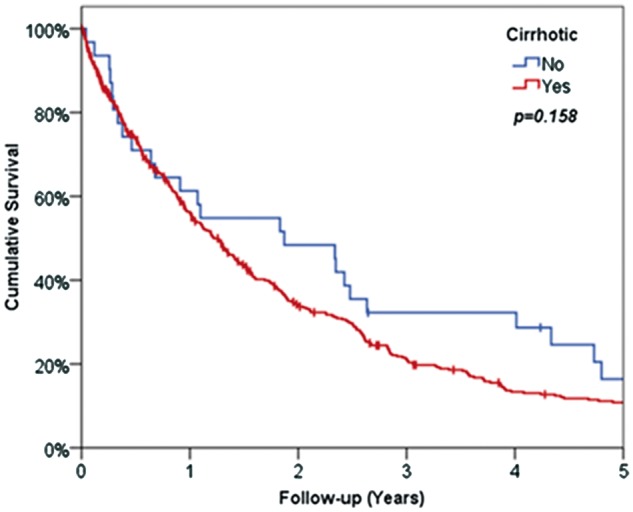
Kaplan-Meier survival curve for patients with cirrhosis and without cirrhosis.

None of the patients from non-cirrhotic cohort received liver transplantation (LT), due to them having large tumour size (69% being ≥5 cm), vascular invasion (16%) or the presence of distant organ metastasis (13%). Despite these differences, survival was not found to differ significantly between the two cohorts (median non-cirrhotic 22 months vs. cirrhotic 15 months, *P* = 0.158, Figure 4), although the statistical power of this analysis was low, on account of the small numbers of non-cirrhotic patients.

#### Comparison between two transplant units

Patient diagnoses differed significantly between the two centres (*P* < 0.001), with the Birmingham cohort being predominantly HCV (73%), whilst the majority of patients from Newcastle had NAFLD (68%). Patient demographics also differed significantly between the sites, with patients from Newcastle being significantly older (*P* < 0.001), with higher BMI (*P* = 0.026), and a higher rate of diabetes (*P* < 0.001), which is likely reflective of the greater proportion of patients at this site with underlying NAFLD. There were also significant ethnic differences (*P* < 0.001), with the Newcastle cohort being almost entirely Caucasian (96%), compared with 75% in Birmingham (Table 6).

**Table 6:** Comparison between all the patients treated at the two transplant centres

	Valid *n*	Birmingham (*n* = 290)	Newcastle (*n* = 197)	*P*-value
**Disease**	**487**			<0.001*
* HCV*		212 (73.1%)	63 (32.0%)	
* NAFLD*		78 (26.9%)	134 (68.0%)	
**Age**^a^	**487**	**60.1 (10.5)**	**68.2 (10.0)**	<0.001*^,^^a^
**BMI**^a^	**336**	**28.6 (6.0)**	**30.1 (6.2)**	0.026*^,^^a^
Gender (Male)	487	237 (81.7%)	153 (77.7%)	0.299
**Ethnicity**	**487**			<0.001*
White		217 (74.8%)	189 (95.9%)	
Asian		64 (22.1%)	5 (2.5%)	
Mixed/Black		9 (3.1%)	3 (1.5%)	
**DM (type 2)**	**487**	**91 (31.4%)**	**127 (64.5%)**	<0.001*
MELD^b^	287	9 (8 - 12)	10 (7–15)	0.328^b^
Albumin^a^	288	38.0 (6.0)	36.5 (5.6)	0.188^a^
**Alcohol excess**	**487**	**59 (20.3%)**	**20 (10.2%)**	0.003*
**Cirrhotic**	**487**	**286 (98.6%)**	**170 (86.3%)**	<0.001*
Location of HCC	387			0.493
Right		173 (60.3%)	64 (64.0%)	
Left		36 (12.5%)	16 (16.0%)	
Both		66 (23.0%)	18 (18.0%)	
Other		12 (4.2%)	2 (2.0%)	
**Largest HCC size (cm)**	**455**			0.014*
<2.0		43 (16.0%)	20 (10.7%)	
2.0–4.9		155 (57.8%)	95 (50.8%)	
5.0+		70 (26.1%)	72 (38.5%)	
Number of HCC	486			0.073
0–2		226 (78.2%)	138 (70.1%)	
3–5		47 (16.3%)	39 (19.8%)	
>5		16 (5.5%)	20 (10.2%)	
AFP^b^	458	27 (6 - 237)	19 (4–179)	0.179^b^
RFA	487	38 (13.1%)	33 (16.8%)	0.296
**TACE**	**487**	**68 (23.4%)**	**97 (49.2%)**	<0.001*
Liver resection	487	10 (3.4%)	7 (3.6%)	1.000
**PEI/alcohol injection**	**487**	**13 (4.5%)**	**0 (0.0%)**	0.001*
Sorafenib	487	20 (6.9%)	10 (5.1%)	0.449
**Liver transplantation**	**487**	**75 (25.9%)**	**26 (13.2%)**	**<0.001**
Median survival (months)^c^	487	16.1 (SE = 1.5)	12.8 (SE = 1.5)	0.927^c^

Data reported as ‘n (%)’, with *P*-values from Fisher’s exact test, unless stated otherwise.

^a^Data reported as ‘Mean (SD)’, with *P*-values from independent samples *t*-tests.

^b^Data reported as ‘Median (Quartiles)’, with *P*-values from Mann–Whitney tests.

^c^Data reported as ‘Kaplan Meier Estimated Rate (SE)’ or ‘Median Survival (SE)’, with *P*-values from log-rank tests.

* Significant at *P* < 0.05.

Of the disease-related factors, rates of alcohol excess (20 vs.10%, *P* = 0.003) and cirrhosis (99 vs. 86%, *P* < 0.001) were both significantly higher in Birmingham. No significant differences in albumin levels, MELD, AFP levels and numbers of HCC were detected between the cohorts. The sizes of HCC were significantly larger in Newcastle patients (*P* = 0.014). Patients from Newcastle were more likely to receive TACE therapy (49 vs. 23%, *P* < 0.001). Overall median survival of patients was similar in the two units (16 vs. 13 months, *P* = 0.927).

#### Comparison as per time frame

We divided the time frame into two periods: from 2000 to 2008 (*n* = 220) and from 2009 to 2012 (*n* = 267), in order to assess whether patient demographics, disease-related factors or outcomes varied over the period of the study. All the factors reported in Table 6 were then compared between the patients in these two time frames. None of the demographic factors were found to differ significantly between the two periods. Of the disease-related factors considered, the only significant difference was in the number of HCCs at presentation, which has reduced significantly over time (*P* = 0.016) with 11% of patients in 2000–8 having more than five HCCs, compared with 5% in 2009–12. As for treatment factors, patients diagnosed in the period 2009–12 were more likely to receive sorafenib (10 vs. 1%, *P* < 0.001), since this treatment only became available in 2008. No significant difference in survival was detected between the periods (*P* = 0.060), with a median of 14 months in 2000–8, compared with 16 months in 2009–12.

## Discussion

HCC remains a common malignancy despite the development of preventative and therapeutic strategies over the past two decades, and overall survival remains extremely poor.^21^^,^^22^ Previous studies have shown that survival in HCC depends on tumour stage, underlying liver function and performance status of the patient.^23^. Worldwide, 50% of HCC is caused by HBV, although in western countries, 30% of HCC were related to HCV.^21^ Other factors noted to increase the risk of cirrhosis and HCC include alcohol excess, diabetes and obesity.^16^^,^^24–26^ Obesity is increasing worldwide and a recent meta-analysis of 11 cohort studies from Europe, the USA and Asia showed that summary relative risks of HCC were 1.17 (95% CI: 1.02–1.34) for overweight and 1.89 (95%CI: 1.51-2.36) for obese individuals, compared with normal-weight individuals.^27^

In our collaborative study, patients in the NAFLD groups were significantly older and more likely to be Caucasian in origin than those with HCV which was also observed by Ascha *et al.*^16^ The findings from a study in the USA demonstrated a significantly higher proportion of females in the NAFLD cohort.^28^ In our UK- based study, we did not detect any differences in gender between the two cohorts, with a high male preponderance observed in both groups. Our study showed that patients with NAFLD tend to have larger tumour size, but lower AFP level compared to those with HCV. NAFLD patients had larger tumour size at diagnosis, probably due to lower rate of detection with ultrasound likely to be limited by presence of central abdominal obesity as well as under-estimation of the degree of fibrosis which can then reduce the rate of surveillance in this cohort of patients.

With the increasing use of fibroscan with larger probes, more patients with NAFLD are being assessed for the presence of significant fibrosis and the debate is still ongoing for performing HCC surveillance in patients with F3 fibrosis. Interestingly, despite having larger tumours, the rates of vascular invasion, lymph node or distant organ metastasis were similar in the two groups. In our cohort, 13% of NAFLD patients were not cirrhotic at the time of HCC diagnosis. In the non-cirrhotic cohort, patients were significantly older with higher diabetes rate and significantly larger tumour size. In this cohort of non-cirrhotic patients, 26% received liver resection but none received LT due to either tumour size outside transplant criteria, vascular invasion or distant organ metastasis

In our study, NAFLD patients were more commonly treated with TACE than the HCV group, whilst the HCV group were significantly more likely to be transplanted. The liver resection rate was low in both of our cohorts because of the underlying degree of liver fibrosis as well as associated co-morbidities in NAFLD cohort. In regard to time line, we found that sorafenib was used more commonly between 2009 and 2012, since the phase 3 clinical trial of sorafenib was published in 2008.^28^

Our patients had comparable rates of transplantation to the previous study performed by Ascha *et al*.^16^ in which 29% underwent liver transplantation and in our study, crude rate of liver transplantation over 14 years of follow up as 21% (101/487 = 21%). In our data, more patients (29.1%) of HCV-HCC were transplanted compared with 9.9% of NAFLD-HCC patients. These difference were likely to be due to unfavourable patient characteristics in NAFLD cohort (older, more likely to be diabetic and higher BMI) as well as larger tumour sizes. Although ∼50% survival at 5 years makes liver transplantation, comparatively, one of the best treatments for hepatocellular cancer, it raises the question as to whether this is an appropriate use of limited organ resources. Utilizing cut-off AFP value as an added selection criterion during LT assessment will hopefully reduce future deaths from post-transplant recurrence.^28^^,^^29^

Furthermore, a proportion of patients with HCV develop severe HCV recurrence in the liver graft post-LT. However, the recent development in newer antiviral therapy with high sustained virologic response rates should also impact on outcome for patients with HCV transplanted for HCC.^30–32^ This should hopefully prevent graft loss from recurrent HCV but we should be cautious regarding HCC recurrence rates in individual treated with newer agents due to a recent study in the pre-transplant population.^33^ In this particular study, 103 patients who received direct acting anti-viral (DAA), 16 (27.6%) developed radiological recurrence of tumour.^33^ NAFLD post-transplant survival rates did not significantly differ from HCV patients but were below 50% at 5 years. The total numbers were small to draw definite conclusions but utilizing cut off AFP values and improved risk stratification of cardiovascular co-morbidity will hopefully improve outcomes in the future.

In our cohort, a much higher proportion of HCV patients were transplanted compared with NAFLD patients and the reasons behind was likely multifactorial including: their younger age at presentation, increased likelihood of being cirrhotic at presentation, the reduced rate of underlying co-morbidities especially cardiovascular risk factors and lower BMI with reduced surgical risk. Due to this lower risk, the HCV patients are also more likely to be allocated livers from donors after circulatory death, widening the pool of available organs for these patients. Furthermore, in terms of tumour biology NALFD patients had larger tumours at presentation although other factors such as number of tumours, extra hepatic spread were similar and AFP values were lower than HCV patients.

When we compared the two units (Birmingham and Newcastle), we found that patients from Newcastle had higher risk baseline demographics (patients were older, higher BMI and more likely to be diabetic) than Birmingham, reflected in fact that the majority of patients from Newcastle having underlying NAFLD rather than HCV. As per tumour characteristics, more patients from Newcastle received TACE treatment, which was attributed to the higher proportion of NAFLD associated HCC patients, and the less favourable patient and tumour characteristics.

There are limitations with our study, one being the retrospective nature of the data collection and analysis. Data were incomplete for some of the variables, most noticeably for MELD and albumin data. It is also important to highlight that these are the data from patients presented to tertiary centre liver transplant units in which patients were managed in a multidisciplinary team, and so further studies are required for presentation of HCC in secondary and primary care. In terms of therapy, we can demonstrate that the majority of patients in both cohorts present with advanced/incurable disease. It is therefore clear that further work is required to improve future risk stratification and early stage diagnosis, irrespective of how HCC epidemiology changes in the future.

## Conclusion

Despite being older with more metabolic risk factors as well as presenting with larger tumours, a significant proportion of patients with NAFLD are able to tolerate loco-regional therapy such as TACE or RFA and have similar overall survival compared to those with HCV with lower tumour burden.

The new era of anti-viral therapies has transformed our care of patients with chronic hepatitis C. These newer agents with excellent sustained virological response may reduce the incidence of HCC in HCV cohort in the near future. However, the overall incidence of HCC is likely to increase as a result of worsening incidence of obesity worldwide with increased incidence of NAFLD. Hence, it is important to understand the nature of HCC in patients with NAFLD in order to plan future surveillance and therapeutic approaches.

## Supplementary Material

Supplementary Data
