# Supplementary material for: Comparing clinical presentations, treatments and outcomes of hepatocellular carcinoma due to hepatitis C and non-alcoholic fatty liver disease
Source: QJM. 2016 Sep 10;110(2):73–81. doi: 10.1093/qjmed/hcw151 (PMC5444673; doi:10.1093/qjmed/hcw151)
Supplement: Supplementary Data [file hcw151_Supp.pdf]

Supplementary Table 1a – Univariable predictors of survival (patient factors)

**Factor**	**HR (95% CI)**	**p-Value**
Disease (NAFLD)	1.08 (0.88 - 1.31)	0.465
**Age**		**0.019***
<55	-	-
55-64	0.94 (0.71 - 1.25)	0.684
65-74	1.14 (0.87 - 1.49)	0.358
75+	1.46 (1.08 - 1.98)	0.013*
BMI		0.424
<25	-	-
25-29	1.20 (0.87 - 1.66)	0.258
30-34	1.28 (0.92 - 1.77)	0.146
35+	1.29 (0.90 - 1.84)	0.173
**Gender (Male)**	0.69 (0.55 - 0.88)	**0.003***
Ethnicity		0.703
White	-	-
Asian	0.95 (0.71 – 1.26)	0.703
Mixed/Black	1.35 (0.60 – 3.04)	0.465
Diabetes Mellitus	0.83 (0.68 – 1.00)	0.055
Alcohol Excess	1.01 (0.77 – 1.31)	0.963
Cirrhotic	1.32 (0.90 – 1.94)	0.159
MELD		0.175
<8	-	-
8-10	1.40 (0.98 – 2.00)	0.065
11+	1.30 (0.90 – 1.87)	0.169
**Albumin**		**0.032***
<35	-	-
35-39	0.91 (0.65 - 1.28)	0.583
40-44	0.71 (0.50 - 1.03)	0.071
45+	0.50 (0.30 - 0.84)	0.008*

Results from univariable cox regression models.
**Significant at p<0.05*

Supplementary Table 1b – Univariable predictors of survival (disease and treatment factors)

**Factor**	**HR (95% CI)**	**p-Value**
Location of HCC		0.843
Right	-	-
Left	0.89 (0.64 - 1.23)	0.490
Both	0.97 (0.73 - 1.29)	0.834
Other	0.78 (0.37 - 1.66)	0.519
**Largest HCC Size (cm)**		**<0.001***
< 2.0	-	-
2.0-4.9	1.85 (1.31 - 2.61)	<0.001*
5.0+	3.31 (2.30 - 4.74)	<0.001*
**Number of HCC**		**0.029***
0-2	-	-
3-5	1.23 (0.95 - 1.59)	0.122
>5	1.54 (1.08 - 2.19)	0.018*
**AFP**		**<0.001***
<5	-	-
5-24	1.12 (0.83 - 1.51)	0.462
25-249	1.66 (1.24 - 2.22)	<0.001*
250+	3.36 (2.49 - 4.55)	<0.001*
**Vascular Invasion**	3.60 (2.75 - 4.73)	**<0.001***
**Lymph nodes**	2.62 (1.43 - 4.78)	**0.002***
**Distant Organ Mets**	1.64 (1.08 - 2.51)	**0.021***
**RFA**	0.48 (0.36 - 0.64)	**<0.001***
**TACE**	0.70 (0.57 - 0.86)	**<0.001***
**Liver Resection**	0.40 (0.23 - 0.72)	**0.002***
Percutaneous ethanol Injection (PEI)	0.52 (0.27 - 1.01)	0.054
Sorafenib therapy	0.98 (0.67 - 1.44)	0.927
**Transplanted**[#]	0.44 (0.32 - 0.62)	**<0.001***

Results from univariable cox regression models.

[#]*Treated as a time-dependent covariate, in order to account for the effect of survivor bias.*

**Significant at p<0.05*
